# Identification of Proteins Implicated in the Increased Heart Rate in ShenSongYangXin-Treated Bradycardia Rabbits by iTRAQ-Based Quantitative Proteomics

**DOI:** 10.1155/2015/385953

**Published:** 2015-12-03

**Authors:** Zhouying Liu, Jian Huang, Youping Huo, Jing Gong, Yinhui Zhang, Cong Wei, Jielin Pu

**Affiliations:** ^1^State Key Laboratory of Translational Cardiovascular Medicine, Fuwai Hospital & Cardiovascular Institute, Chinese Academy of Medical Sciences and Peking Union Medical College, 167 Bei-Li-Shi Road, Xi-Cheng, Beijing 100037, China; ^2^Integration of Traditional and Western Medical Research Academy of Hebei Province, Shijiazhuang 050035, China

## Abstract

The present study tries to identify proteins implicated in bradycardia rabbits in hearts after ShenSongYangXin (SSYX, a traditional Chinese medicine) treatment. Eighteen adult rabbits were randomly assigned to three groups: sham, model, and SSYX treatment groups. Heart rate was recorded in rabbits and proteins were isolated from ventricular muscle. We used isobaric tags for elative and absolute quantitation (iTRAQ) coupled with two-dimensional liquid chromatography-tandem mass spectrometry to identify altered proteins after SSYX treatment. The heart rate decreased after six weeks due to the injury of the sinoatrial node in the model group. This effect was partially reversed by 4-week SSYX treatment. A total of a2988 proteins were quantified by performing the iTRAQ-based experiments. Of these, 86 proteins were differentially expressed according to our criteria (42 upregulated and 44 downregulated). The identification of key proteins implicated in the treatment of bradycardia could serve as a foundation to better understand and further explore the molecular mechanism of SSYX treatment.

## 1. Introduction

Bradycardia is a condition in which pulse rate is below 60 beats per minute (bpm). Coronary artery disease patients and elderly people are at a great risk of developing the abnormally slow heart rate [1]. Currently available drugs (e.g., atropine, dopamine, isoproterenol, and epinephrine) treating bradycardia are temporizing measures only in emergency settings. If the patient does not respond to drugs, temporary or permanent cardiac pacemaker is probably indicated [2]. However, the cost of pacing put a huge financial burden on the family. Consequently, an effective drug aiming at increasing heart rate for a long term is in urgent demand.

Traditional Chinese medicine has been used to treat arrhythmia for hundreds of years and ShenSongYangXin (SSYX) is one of such medicines. It is a product consisting of 12 ingredients including Panax ginseng, dwarf lilyturf tuber, and Nardostachys root. Whole cell patch clamping experiments revealed that SSYX could block multiple ion channels [3]. Preliminary studies also suggested that SSYX was effective in reducing ventricular premature beat and treating bradycardia [4, 5]. In addition, the latest results from a randomized, double-blind, placebo-controlled multicenter trial demonstrated that SSYX is effective for patients with bradycardia [6]. Recently, we explored the gene expression profiling of cardiac atrium after SSYX treatment [7]. However, it has been a mystery about the protein alterations in cardiac ventricle after SSYX treatment. Therefore, we established an animal model of bradycardia and explored the protein alteration of heart after SSYX treatment. Our work will provide new insights into the molecular mechanisms of SSYX.

## 2. Materials and Methods

### 2.1. Bradycardia Model

Eighteen adult rabbits (*Oryctolagus cuniculus*) with mean body weight of 2.5 ± 0.5 kg were used for the study. The experimental protocol was performed in accordance with the Guide for the Care and Use of Laboratory Animals (NIH Publication number 85-23, revised 1985) and The ARRIVE [8]. The Care of Experimental Animals Committee of the Chinese Academy of Medical Sciences and Peking Union Medical College approved the procedures for the care and treatment of animals. The animals were randomly divided into three groups (*n* = 6 in each group): sham, model, and model plus SSYX (SSYX) groups. Sterilized cotton bud with formaldehyde (37%, SCRC) was fixed on the wall of the right atrium, near the entrance of the superior vena cava until heart beat decreased 25–35% [9]. The time span of the process was ranged from 20 second to 3 minutes. Continuous monitoring by electrocardiogram for two weeks confirmed the slow heart rate. Then, purified water was administered orally to model group while dry powder of SSYX (220 mg/kg/d) dissolved in purified water was administered to SSYX group. Lead II was used to monitor the electrocardiogram, once every week for 4 weeks. The procedures in sham group were similar to model and SSYX group except that formaldehyde was replaced by purified water. RR, P, PR, QRS, QT, and QTc were calculated before operation (baseline) and 6 weeks later, respectively.

### 2.2. Histopathology

Animals were sacrificed 6 weeks later. Hearts of the animals were isolated and perfused with purified water. Atria and ventricle of the heart were immediately frozen in liquid nitrogen and then stored at −80°C until use for protein isolation. A piece of each left ventricle was fixed with 5% glutaraldehyde. The Hematoxylin and Eosin (H&E) and Masson's Trichrome were performed. Images were obtained with Image-Pro Plus 6.0 (Media Cybernetics Inc., USA). Three fragments of every heart were randomly chosen for further analysis using electron microscope (JEOL-1230, 80 kv, Japan).

### 2.3. Protein Preparation and iTRAQ Labeling

Protein preparation from rabbits was performed following the published method [10] with some modifications. Briefly, ventricles from three rabbits were pooled and homogenized in 5 volumes (v/w) of isolation buffer (0.3 M sucrose, 10 mM Hepes-Na, pH 7.0, 0.5 mM EDTA, 2 mM PMSF, and 1 : 1000 diluted Protease Inhibitor Cocktail (Sigma P8340)) using a Dounce glass/Teflon homogenizer. Centrifugations were carried out twice to discard nuclear and cell debris, 800 ×g, 4°C for 10 min. The supernatant was then collected and stored at −80°C.

To minimize variations attributable to individual rabbits and maximize differences attributable to their genotype, each experiment was performed with RNA pooled from three ventricles. Equal amounts of protein (75 *μ*g) from each pooled sample were digested with trypsin (0.5 *μ*g/*μ*L) at 37°C for 16 h and labeled with a unique iTRAQ reagent (114 for model group, 116 for SSYX group). Labeled samples were pooled and dried in a vacuum centrifuge.

### 2.4. iTRAQ Proteomic Analysis

The labeled dried peptides were dissolved in Mobile phases A (2% acetonitrile (ACN), pH 10.0). Then samples were loaded on the reversed phase (RP) column (Agela, 5 *μ*m, 150 Å, 4.6 × 250 mm) and separated on an L-3000 HPLC system (Rigol, China) at a flow rate of 1 mL/min. Mobile phase A consisted of 2% ACN and mobile phase B consisted of 98% ACN. Both of them adjusted pH to 10.0 using NH_3_·H_2_O. The gradient used was described as follows: 5–8% B, 2 min; 8–18% B, 11 min; 18–32% B, 9 min; 32–95% B, 1 min; 95% B, 1 min; 95–5% B, 2 min. The temperature of Column Oven was set as 60°C. Fractions were collected every minute and then dried in a vacuum centrifuge. Forty fractions were collected and desalted. Then, fractions were combined into twelve fractions and vacuum-dried until analyzed by LC/MS/MS.

After being dissolved in 0.2% FA and 5% methanol, the dried tryptic peptides were loaded and trapped on a precolumn (C_18_, 100 *μ*m × 20 mm, 5 *μ*m particle size) and then separated on an analytical column (C_18_, 75 *μ*m × 150 mm, 3 *μ*m particle size). Peptides were eluted from the C_18_ analytical column with 40 min gradient at a flow rate of 350 nL/min on Eksigent Ultra HPLC (AB Sciex). The MS conditions for Triple TOF 5600 were set as follows: the spray voltage was set to 2.5 kv and the temperature of heater was 150°C. The MS scan range was set at 350 to 1250 m/z and the MS/MS scan range was 100–1500 m/z. Data dependent acquisition was performed and top 50 precursor ions were selected to fragment using CID (collision induced dissociation). The collision induced dissociation energy was automatically adjusted by the rolling CID function.

### 2.5. Database Search and Bioinformatics

The resulting MS/MS data were then compared against data in the NCBI database (Rabbit.protein-20150201) using ProteinPilot Software Beta (AB, USA, version 4.5). For protein identification and quantification, peptide mass tolerance and fragment tolerance were each set at 0.3 Da. Only one missed tryptic cleavage was allowed. The false positive rates were controlled below 1%. The following criteria were used to select differentially expressed proteins: (1) proteins including at least one unique high-scoring peptide; (2) *P* value < 0.05; and (3) fold-changes needed to be greater than 2 or less than 0.5. The UniProt knowledge base (Swiss-Prot/TrEMBL, http://www.uniprot.org/) and gene ontology (GO) database were applied to further classify these differentially expressed proteins.

### 2.6. Western Blot

Samples were prepared in SDS sample buffer, separated on 10% SDS gel, and transferred to 0.45 *μ*m PVDF membranes. The membranes were incubated with primary antibodies for RyR2 and SERCA2 and with AP-conjugated secondary antibodies. Proteins were detected by BCIP/NBT method following the instruction for the western blot kit.

### 2.7. Statistical Analysis

Electrocardiogram data were expressed as mean ± SEM. Two-way ANOWA was used to test difference of basic parameters between groups. Independent sample *t*-test was used to estimate difference between groups, with *P* < 0.05 considered as significant. Analyses were performed with SPSS 17.0 (SPSS lnc. Chicago, IL).

## 3. Results

### 3.1. Effect of Long-Term SSYX Treatment on Slow Heart Rate

**Figure 1:**
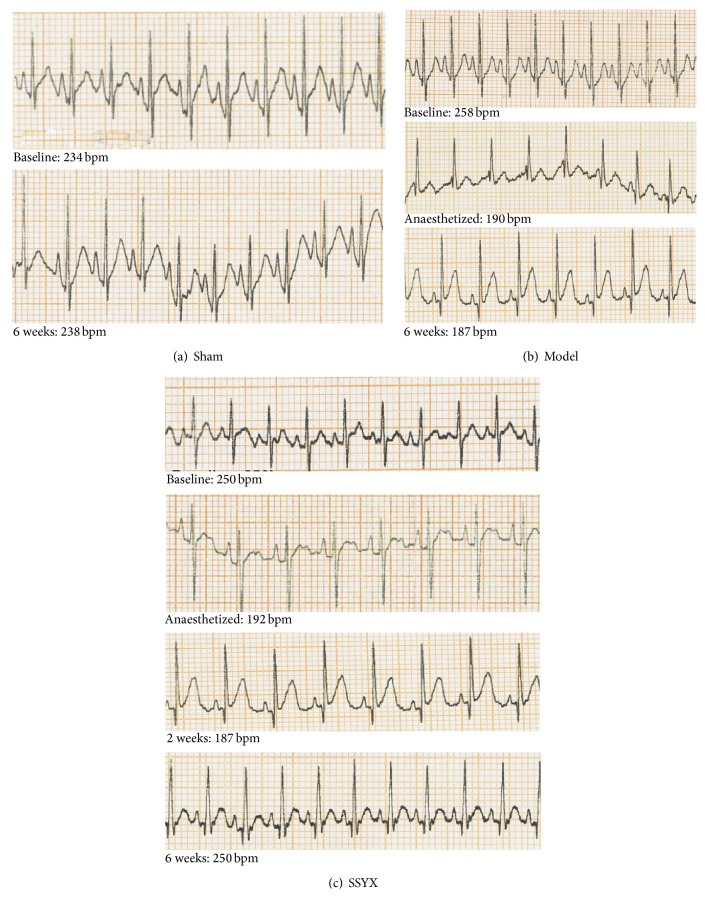
SSYX effects on cardiac electrical activity in anesthetized rabbits. Representative ECG recording (lead II) obtained in one rabbit from sham (a), model (b), and SSYX group (c) under baseline conditions (previous to operation), anaesthetized, after 2 weeks and 6 weeks of treatment.

Representative ECG recordings of sham, model, and SSYX-treated rabbits were displayed and analyzed in Figure 1 and Table 1, respectively. No significant difference was observed among basic heart rate (baselines) of three groups (*P* > 0.05). It was obvious that chemical injury of sinoatrial node reduced the mean heart rate by 32% (sham group: 219 ± 14 bpm; model group: 148 ± 12 bpm, *P* < 0.05, *n* = 6 in each group). The decreased heart rate was partially restored by long-term (4-week) SSYX treatment (220 mg/kg·d, SSYX group: 228 ± 14 bpm, *P* < 0.05), while other parameters included atrial, atrioventricular, and ventricular conduction and ventricular repolarization were not significantly altered due to the unmodified P, PR, QRS, and QT interval (Table 1).

### 3.2. Effects of Long-Term SSYX Treatment on Left Ventricle

**Figure 2:**
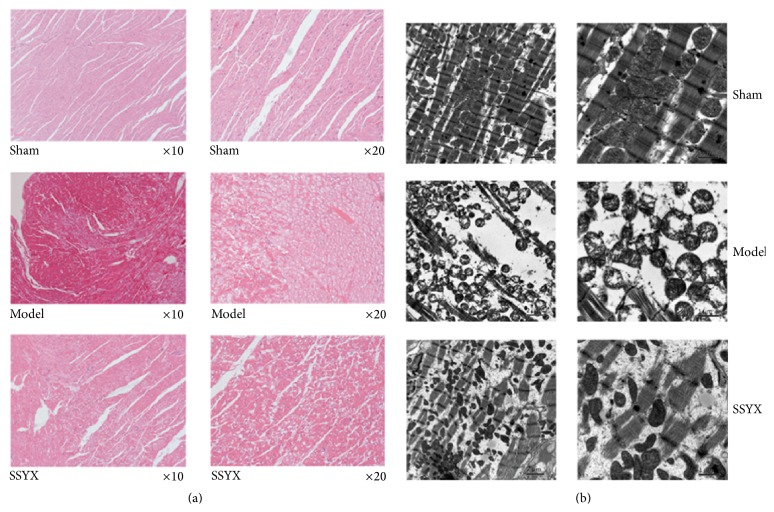
Images obtained from light and electron microscope. (a) Light microscope of left ventricle (HE staining) from sham, model, and SSYX groups. The magnification is ×10 (left) and ×20 (right). (b) Electron microscope of left ventricle from three groups. The magnification is ×5000 (5 k, left) and ×12000 (12 k, right).

As shown in Figure 2(a), serious vacuolar degeneration and fibrosis can be seen in model group while these pathological alterations were alleviated in SSYX group. Electronic images also displayed myofilament degeneration, obvious contraction bands, and increased swollen mitochondria. These mitochondria were characterized with vacuolar degeneration and disrupted mitochondrial cristae (Figure 2(b)). Myofilament degeneration and mitochondrial vacuolar degeneration were relieved and disrupted mitochondrial cristae were restored by SSYX treatment.

### 3.3. Effects of Long-Term SSYX Treatment on Cardiac Proteins

To further explore the molecular mechanism of SSYX treatment, we applied iTRAQ to quantify proteins of ventricle. A total of 2988 proteins were detected. After comparing model and SSYX group, 86 altered proteins (44 downregulated proteins and 42 upregulated proteins) were identified after SSYX treatment according to our criterion (*P* value < 0.05, and fold change >2 or <0.5).

**Figure 3:**
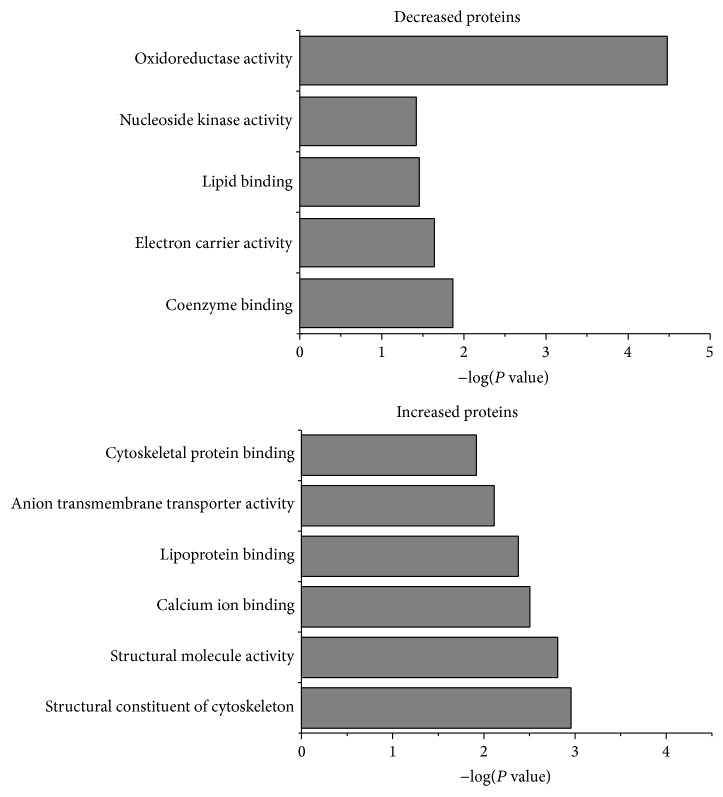
Effects of long-term SSYX treatment on cardiac proteins. After GO analysis (biological process), decreased proteins (up) were involved in oxidoreductase activity and electron carrier activity while increased proteins (down) were involved in calcium ion binding, structural constituent of cytoskeleton, and structural molecule activity.

A complete list of them was attached in supplementary table and classified among three categories: molecular function, biological process, and cell component (Supplementary Figure; see Supplementary Material available online at http://dx.doi.org/10.1155/2015/385953). After GO analysis (biological process), altered proteins were involved in calcium ion binding, structural constituent of cytoskeleton, structural molecule activity, oxidoreductase activity, electron carrier activity, and so forth (Figure 3). It was particularly worth noting that calcium signaling became more active after SSYX treatment. Three kinds of calcium channels were increased including calcium release channel (RyR2), SR Ca^2+^-ATPase (SERCA2), and voltage-dependent anion-selective channel (VDAC). RyR2 locate at sarcoplasmic reticulum (SR) and release SR calcium to cytoplasm. SERCA2 reuptake the released calcium into SR while VDAC transport calcium in mitochondria [11]. The increased protein level of them indicated the improved activity of calcium handing of SR and mitochondria. In addition, calcium-associated proteins were increased, such as SLC25A12, S100A8, S100A9, SPTA1, THBS1, and STAT1, and all these results demonstrated that calcium homeostasis plays a critical role in SSYX treatment. The other increased group of protein was structural proteins of heart, such as TLN1, SORBS2, SPTA1, and SPTB, which implied that the destroyed structure of myocardium were to some extent restored. This result was also in agreement with images from electron microscope.

The decreased proteins were involved in glycolysis/gluconeogenesis, fatty acid, fatty acid metabolism, and so forth, which means SSYX inhibited the metabolism of myocardium.

### 3.4. Verification of Differentially Expressed Proteins by Western Blot

**Figure 4:**
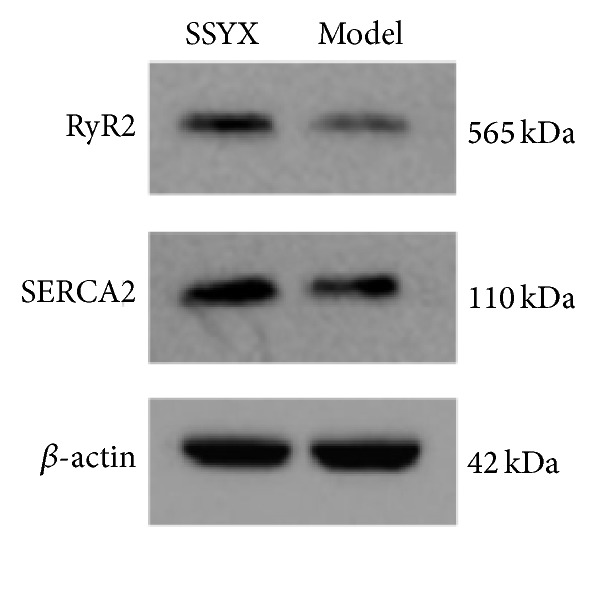
Expression of RyR2 and SERCA2 after SXSM treatment by western blotting. The results of western blotting were from a representative of three repeated experiments.

Western blot was performed to confirm the results from iTRAQ. Considering the biological function, we selected two increased proteins:* RyR2* and* SERCA2*. *β*-actin was also detected as an internal control. As demonstrated in Figure 4,* RyR2* and* SERCA2* increased after four weeks of treatment with SSYX. Therefore, the trend of quantification verified by western blot was in agreement with that detected by iTRAQ.

## 4. Discussion

iTRAQ-based quantitative proteomics have been applied to explore protein alterations in cardiovascular diseases such as atrial fibrillation, hypertension, and heart failure [12–16]. Both acute and chronic experiments of cardiomyocytes using iTRAQ were reported, as well [17, 18]. The molecular mechanisms of SSYX administration have principally been unknown. The present study demonstrates that SSYX is effective in treating bradycardia by altering proteins involved in calcium ion related proteins, structure proteins, oxidoreductase activity, and electron carrier activity.

According to iTRAQ result, calcium ion related proteins (including calcium channels and calcium binding proteins) and structure proteins were the most stimulated by SSYX. On the one hand, increased RyR2 could release more luminal Ca^2+^ into cytoplasm. Thus the enhanced Ca^2+^ transients would lead to strengthened contraction and inhibition of the negative effect of intra-SR Ca^2+^ overload. On the other hand, Ca^2+^-ATPase (encoded by SERCA2) and voltage-dependent anion channel were also enhanced, which indicated that released cytoplasm Ca^2+^ from SR was reuptaken into SR or transported into mitochondria.

The increased SR Ca^2+^ uptake and RyR2 activity will result in a more synchronous, faster, and abbreviated Ca^2+^ release that was required for maintaining faster heart rates [19]. In addition, they reduced the concentration of cytoplasm and thus inhibited the negative effect of calcium overload. The swollen mitochondria were the same as myocytes after calcium overload [20]. Moreover, severe cellular damage (myofilament degeneration), which may be initiated by calcium overload, could also be observed in ultrastructure. All these alterations were alleviated by SSYX, which confirmed that calcium overload was relieved by SSYX. It was reasonable that ATP was declined when calcium overloaded. Despite the reduced electron carrier proteins after SSYX, mitochondria might produce more ATP considering the restored ultrastructure of mitochondria.

Another discovery was that decreased proteins were involved in glycolysis/gluconeogenesis, fatty acid, fatty acid metabolism, and so forth; the inhibited substance metabolism reduced the consumption of ATP and contributed to the recovery of muscle contraction.

### 4.1. Conclusions

Our bradycardia model showed that long-term SSYX treatment increased heart rate and altered proteins of ventricle. It increased the expression of proteins related to calcium ion homeostasis, substance metabolisms, and constituent of cytoskeleton. Increased RyR2, SERCA2, and VDAC restored calcium ion homeostasis and enhanced cardiac function. Both glycometabolism and lipid metabolism were inhibited after SSYX treatment. In addition, western blot also confirmed increased RyR2, SERCA2, which is inconsistent with results from iTRAQ. These data provide insights for the future study of SSYX.

## Supplementary Material

Supplementary table displayed a complete list of altered proteins between model and SSYX group by iTRAQ. Supplementary figure showed altered proteins between model and SSYX group. These altered proteins were classified among three categories: molecular function (A), biological process (B), and cell component (C).

## Figures and Tables

**Table 1:** ECG parameters in anesthetized rabbit from sham, model, and SSYX groups.

	HR, bpm		P, ms		PR, ms		QRS, ms		QT, ms		QTc, ms	
	Baseline	Week 6	Baseline	Week 6	Baseline	Week 6	Baseline	Week 6	Baseline	Week 6	Baseline	Week 6
Sham	245 ± 10	249 ± 14	40 ± 0	40 ± 0	63 ± 3	63 ± 3	23 ± 3	23 ± 3	170 ± 4	170 ± 4	102 ± 3	103 ± 2
Model	244 ± 5	148 ± 12^*※*^	40 ± 0	40 ± 0	62 ± 1	60 ± 0	22 ± 2	25 ± 3	163 ± 3	185 ± 11	105 ± 2	93 ± 6
SSYX	258 ± 9	228 ± 14^*∗*^	40 ± 0	37 ± 3	63 ± 3	56 ± 3	30 ± 4	27 ± 4	148 ± 4	160 ± 12	97 ± 2	92 ± 4

Data expressed as mean ± SEM. *n* = 6 in each group. Electrocardiogram parameters obtained in anesthetized rabbit from sham, model, and SSYX groups before treatment (baseline) and after 6 weeks of treatment. HR: heart rate; P: P wave duration; PQ: PQ interval; QRS: QRS complex duration; QT: QT interval; QTc: corrected QT interval.  QTc = QT/2 √ (RR/100).

^*※*^ *P* < 0.05 versus sham group.

^*∗*^ *P* < 0.05 versus model group.
