# Supplementary material for: Identification of Proteins Implicated in the Increased Heart Rate in ShenSongYangXin-Treated Bradycardia Rabbits by iTRAQ-Based Quantitative Proteomics
Source: Evid Based Complement Alternat Med. 2015 Dec 3;2015:385953. doi: 10.1155/2015/385953 (PMC4685072; doi:10.1155/2015/385953)
Supplement: Supplementary file 1 — Supplementary table displayed a complete list of altered proteins between model and SSYX group by iTRAQ. Supplementary figure showed altered proteins between model and SSYX group. These altered proteins were classified among three categories: molecular function (A), biological process (B), and cell component (C). [file 385953.f1.pdf]

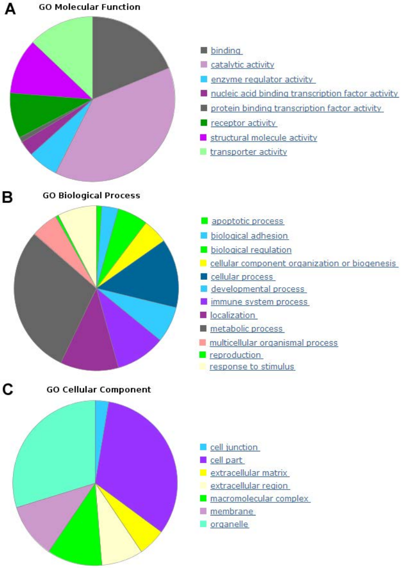
A
GO Molecular Function
binding
catalytic activity
enzyme regulator activity
nucleic acid binding transcription factor activity
protein binding transcription factor activity
receptor activity
structural molecule activity
transporter activity
B
GO Biological Process
apoptotic process
biological adhesion
biological regulation
cellular component organization or biogenesis
cellular process
developmental process
immune system process
localization
metabolic process
multicellular organismal process
reproduction
response to stimulus
C
GO Cellular Component
cell junction
cell part
extracellular matrix
extracellular region
macromolecular complex
membrane
organelle
